# Cheetahs discriminate familiar and unfamiliar human voices

**DOI:** 10.1038/s41598-018-33971-1

**Published:** 2018-10-19

**Authors:** Maël Leroux, Robyn Shelia Hetem, Martine Hausberger, Alban Lemasson

**Affiliations:** 1 Univ Rennes, Normandie Univ, CNRS, EthoS (Éthologie animale et humaine) - UMR 6552, Paimpont, F-35380 France; 2 University of the Witwatersrand, School of Animal, Plant and Environmental Sciences, 1 Jan Smuts Avenue, Braamfontein, 2000 South Africa; 3 CNRS, Univ Rennes, Normandie Univ, EthoS (Éthologie animale et humaine) - UMR 6552, F-35380 Paimpont, France

## Abstract

Domestic species can make the distinction between several human sub-groups, especially between familiar and unfamiliar persons. The Domestication hypothesis assumes that such advanced cognitive skills were driven by domestication itself. However, such capacities have been shown in wild species as well, highlighting the potential role of early experience and proximity with humans. Nevertheless, few studies have been focusing on the use of acoustic cues in wild species and more comparative studies are necessary to better understand this ability. Cheetah is a vocal, semi-social species, often hand raised when captive, making it therefore a good candidate for studying the ability to perceive differences in human voices. In this study, we used playback experiments to investigate whether cheetahs are able to distinguish between the voices of their familiar caretakers and visitors. We found that cheetahs showed a higher visual attention, changed activity more often and faster when the voice was familiar than when it was unfamiliar. This study is the first evidence that wild felids are able to discriminate human voices and could support the idea that early experience and proximity to humans are at least as important as domestication when it comes to the ability to recognize humans.

## Introduction

The ability to discriminate between familiar and unfamiliar humans (e.g. owner, trainer, caretaker), on the basis of visual (pig (*Sus scrofa domesticus*)^1^, cow (*Bos Taurus*)^2,3^, lamb (*Ovis aries*)^4^, horse (*Equus caballus*)^5,6^, dog (*Canis lupuis familiaris*)^7^, cat (*Felis catus*)^8^), olfactory (horse^9^) or acoustic cues (pig^10^, horse^6,11^, dog^12^) is well known in domestic animal species. A common hypothesis is that domestication has driven the selection of advanced cognitive abilities and multi-modal sensitivity to interact with humans^13–15^. However, several studies on visual perception have shown that wild animals that experience interacting with humans positively (e.g. food provisioning) or negatively (e.g. repelling) are also able to discriminate between familiar and unfamiliar persons (e.g. dolphins (*Delphinidae*)^16,17^, crow (*Corvus brachyrhychos*)^18^, magpie (*Pica pica*)^19^). Beyond a possible domestication effect, the simple relative proximity with humans may thus provide an opportunity for such learning with an obvious interest in terms of survival value^20^.

Nevertheless, a few studies have demonstrated the ability of non-domestic animals to discriminate sub-groups of humans on the basis of non-visual cues, particularly on the basis of voice recognition, in species both phylogenetically more (e.g. elephant (*Loxondota Africana*)^21^, crow (*Corvus corone*)^20^) and less (e.g. non-human primates^22–24^) distant from humans. The phylogenetic proximity of species, and hence similarity of vocal repertoires, may facilitate the development of the required skills to perceive and decode heterospecific voices (see for example individual voice recognition and message decoding in non-human primates^22,23^). Yet several animals, including typical vocal and non-vocal communicants (e.g. baboon (*Papio hamadryas ursinus*) - ungulates^24^, Günther’s dik-dik (*Madoqua guentheri*) – Go-away bird (*Corythaixoides leucogaster*)^25^, squirrel (*Sciurus vulgaris*) – jay (*Garrulus glandarius*)^26^), rely on a broad range of other species’ calls to take decisions in their daily life (e.g. reactions to heterospecific alarm calls to protect against predators). Associative learning may thus be a common process explaining that wild and domestic animals may develop the ability to perceive and process heterospecific acoustic signals, decoding these signals at the appropriate level (general reaction to predators, discrimination of groups or individuals when pertinent).

Overall, more comparative studies are needed, notably on non-visual modalities, to better understand the relative importance of daily human-animal interactions on the ability of captive wild animals to use human cues. For example, a case of vocal innovation was found in a captive group of African guenons (*Cercopithecus c*. *campbelli*)^27^. Animals started to produce a new alarm call type, never recorded in the wild, to alert about the approach of some humans (unfamiliar ones or familiar ones providing invasive care). Domestication is thus not the only process by which the ability to use human cues can develop. To our knowledge, no study has ever tested the ability of wild felids to discriminate between different human voices, an ability found in their domestic cousins^28^.

In the present study, we tested the ability of captive cheetahs (*Acinonyx jubatus*) that had early and regular contacts with humans to discriminate familiar and non-familiar human voices. Sound perception and production plays an important role in cheetahs (social life and prey hunting). Cheetahs are felids with a semi-social (solitary females and social male coalitions) system^29^, a varied vocal repertoire^30,31^ and supposedly a comparable audiogram to cats (e.g. Broad hearing frequency range: 48 Hz–85 kHz; as frequency increases the threshold SPL decreases to a minimum between 1 and 10 kHz and the value of the minimum is around −20 dB SPL)^32,33^. According to Volodina^31^ and Smirnova *et al*.^30^, their vocal repertoire is composed of 8 calls produced in a context specific way and that can grade into each other. The meow is the most common vocalization in cheetahs as it is in their domestic relatives^30^. It is produced across context and encodes both sex and identity of the caller^30^. On the contrary, growls, hisses and howls are specific to offensive contexts while chirrs are specific to mating and purrs are produced mainly during human contact^30,31^. Cheetahs are commonly found in captivity where they are often hand-raised and human trained^34^. Here, we tested whether hand-raised male cheetahs could distinguish handlers’ voices from other humans’ (tourists of comparable age and sex) voices.

## Methods

### Study site and animals

Between February and April 2017, eleven adult male cheetahs (aged 8.4 ± 4.3 years old) were tested in the South African education center Cheetah Outreach (CO) in Stellenbosch. All tested individuals were at least the second generation born in captivity and hand-raised. They arrived at CO few months after birth, between 2005 and 2015, and were therefore familiar with the handlers for at least two years. The center was open to the public every day between 10:00 and 17:00, offering views of cheetahs during this time. Cheetahs were housed in outdoor enclosures (2000 ± 400 m^2^) including a wooden shelter filled with a litter of straw, an open fabric shelter and a trough. Subjects were fed before the arrival of the public at 9:00 by the handlers who brought a stainless steel bowl filled with a mixture of chicken, rabbit, pork and horse meat inside the enclosure. They had ad libitum access to water. They also had various dietary enrichments given by handlers during the day (pieces of chicken, balls of icy blood etc.). Individuals were housed in stable groups of related males (dyads or tryads) with the exception of two adult males who were kept alone. All individuals had visual and auditory contact with each other. Tactile contact was possible across fences for adjacent groups and groups changed enclosure on a regular basis in order to allow interactions between different groups. Cheetahs had four different types of contact with humans: (1) feeding as mentioned above, (2) transfers between enclosures which were carried out, on a daily basis, with the individual held on a lead by two handlers, (3) “enrichment” in which one handler and one volunteer interacted with at least one cheetah per day for approximately thirty minutes. The identities of volunteers and handlers in charge of transfers and enrichments changed every day so that all animals get used to all humans. Interaction included brushing, petting or simply sitting nearby to allow the cheetah the choice of initiating contact. Finally, cheetahs were exposed to (4) “encounters” with the public for “public awareness”, these encounters took place under the supervision of a handler (one of 14) and a volunteer (one of 20) in charge of visitors’ safety. In groups of up to five individuals, the public could pet the cheetah in its enclosure. Six individual cheetahs were exposed to the public encounters, these were changed daily although two cheetahs were chosen more frequently. All animals were naive to playback experiments.

### Playback experiments

Cheetahs were confronted with different categories of humans: (1) handlers, who were very familiar to the cheetahs as they had frequently interacted with them for many rewarding activities such as feeding, (2) volunteers, who were familiar to the cheetahs as they interacted with them for short periods (less than a year) primarily during secondary activity such as enrichment and encounters and (3) visitors, who were unfamiliar to the cheetahs as novel tourists visited the center and interacted with the cheetahs for only a few minutes each day. Since we wanted to test fine discrimination, i.e. individual voices, we exposed the cheetah to two stimuli, namely that of a familiar handler and an unfamiliar visitor. Volunteer voices were not taken into account since they were at CO for less than a year and did not take part in feeding or relocating activities. Eleven familiar handler and eleven unfamiliar visitor voices were recorded, which consisted of eight female and three male voices per group. Each individual cheetah was tested twice, once with the voice of a handler and, on a separate occasion, with the voice of an unfamiliar visitor. The handler’s voice played to each cheetah was selected based on observed handler-cheetah interactions, with the most familiar relationships being preferred. The allocation of unfamiliar voices was done semi-randomly (i.e. had to match the sex of the familiar voice used for the same cheetah and to match approximately the age of the familiar person which ranged from 20 to 60 years).

Each individual cheetah was therefore tested using a single “familiar voice” stimulus and a single “unfamiliar voice” stimulus pronouncing the same sentence: “I’ll need a back-handler here”, chosen from pilot observations on handlers’ routines. This sentence was commonly used during different routine procedures such as feeding or transferring the cheetahs. All human voices were recorded in the same silent room (to prevent biases due to background noises), using a directional microphone (Sennheiser® K6/ME66) connected to a portable stereo digital recorder (Marantz® PMD661MKII, sampling frequency = 44100 HZ, resolution = 16 bits,.WAV file format). All the recorded persons were English-speakers, the intonation and the pronunciation were standardized as much as possible by having them first listen to a template and re-recording them to reach the requested prosody. Mann-Whitney U tests were also performed to verify (1) that the length of stimuli did not differ between familiar and unfamiliar voices (respectively 1.36 ± 0.04 s and 1.40 ± 0.07 s (mean ± standard error); Mann-Whitney *U* test, N1 = N2 = 11, *U* = 56.5, *P* = 0.82) and (2) that fundamental frequencies of stimuli did not differ between familiar and unfamiliar voices (respectively 290.5 ± 13.7 Hz and 267.5 ± 19.1 Hz (mean ± standard error); Mann-Whitney *U* test, N1 = N2 = 11, *U* = 48, *P* = 0.37). Voices were played back using a loudspeaker (Sanha® JLH-202D). The volume of the loudspeaker was standardized and the intensity of each acoustic stimuli was adjusted using the Audacity® software to reach 80 db at 20 m (measured using a Phonic® PAA3 decibelmeter) to match the intensity measured during handling routines.

The playback trials started after a six week period of habituation to the experimenter and his recording and playback (with no sound played) equipment. The position of the loudspeaker was standardized, i.e. at the door of the enclosure, making credible the presence of a person. The observer was placed close to the loudspeaker. The playback was performed early in the morning (i.e. between 7:30 and 8:30), before the park was opened to public or staff in order to avoid incongruence or distraction and to standardize the context preceding the playback. Each stimulus (individual human voice) was used only once during the experiment to avoid pseudo-replication, as cheetahs in neighbouring enclosures could also hear the speaker. The order of the stimuli was randomized and we started the experiment with the familiar stimulus for 50% of the subjects and with the unfamiliar stimulus for the remaining 50%. The interval between familiar and unfamiliar playbacks was 6 ± 3 days. Mock experiments (i.e. installation without playing the voice) were carried out on a regular basis in parallel to prevent anticipation (i.e. every two days). The subject cheetah was socially isolated on the eve of the experiment to avoid the influence of a conspecific during the playback and to leave a sufficient period for the habituation to the isolation (animals of this park were regularly subjected to these periods of isolation as part of standard management practices).

The stimulus was played at least five minutes after the experimenter installed the equipment and only when the cheetah was calm (walking, sitting or lying down), did not look towards the loudspeaker or observer and was at a controlled distance from the loudspeaker (~20 m). The temperature ranged between 15 and 20 °C at 8:00 and the wind was consistently less than 5 km/h to avoid potential disturbance of the perception of the stimulus. Apart from the experimenter, no other human was visible by the subject at the time during the playback trial. The individual cheetah’s response was filmed with a camcorder (Sony® Handycam DCR-SX30).

### Data analysis

We defined a reaction as any behavioural change that occurred within the maximum time period of 150 s. Thus, the latency of the first gaze towards the loudspeaker and the total duration of gaze towards the loudspeaker, as well as the latency of the first change of activity (i.e. change in posture and/or locomotor movement) were compared between the familiar and unfamiliar voice playbacks. Individuals who did not exhibit any behavioural change were assigned the maximum latency score of 150 s. Nonparametric Wilcoxon signed-rank tests were then used on Statistica software version 13 to compare the behaviours of subjects in the 150 s following the playback of a familiar *versus* unfamiliar voice. In addition, the number of individuals showing a reaction was compared between familiar and unfamiliar voices using Binomial tests on Statistica software version 13.

To control for the reliability of our video rating, a second blind viewing (with the sound switched off) of 25% of the dataset (half familiar and half unfamiliar voice trials randomly chosen) was conducted by a naive observer. The comparison revealed a 99% inter-observer agreement (Kendall’s concordance coefficient: *W* = 0.97, *P* = 0.0006).

All results are presented as: Mean ± Standard Error.

## Results

**Figure 1:**
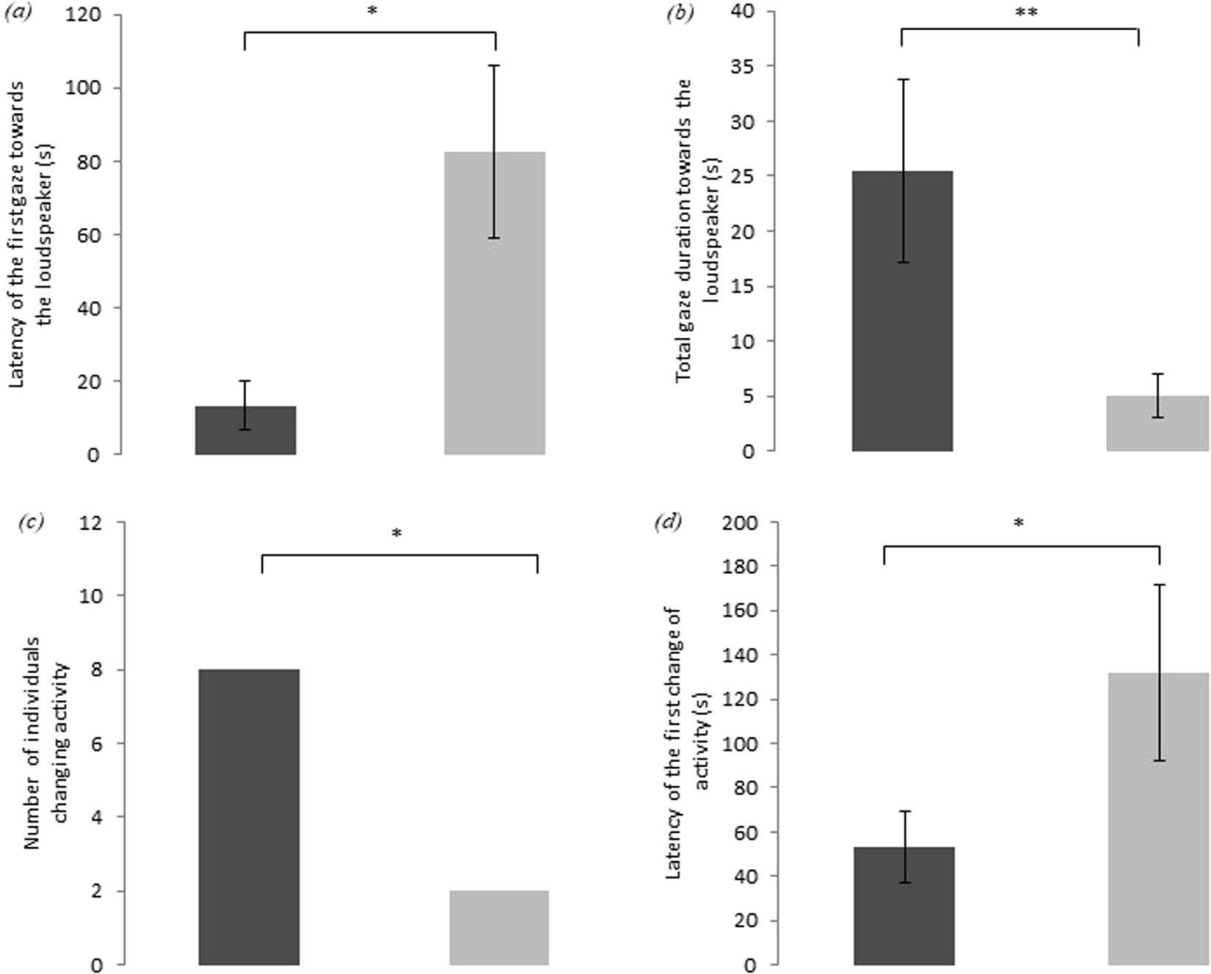
(**a**) Latency of first gaze towards the loudspeaker, (**b**) Total gaze duration toward the loudspeaker, (**c**) Number of individuals changing activity and (**d**) Latency of the first activity change following a familiar (dark grey) or an unfamiliar (light grey) voice playback (a, b and d: mean ± standard error, Wilcoxon signed-rank test; c: Binomial test; *P ≤ 0.05, **P ≤ 0.01).

All 11 cheetahs tested looked at the loudspeaker if the voice was familiar while only five out of 11 looked at it if the voice was unfamiliar. The number of cheetahs who looked at the speaker for the familiar voice and did not look at it for the unfamiliar voice was significantly higher than the reverse (Binomial test, *P* = 0.016). In addition, cheetahs looked at the loudspeaker six times faster after a familiar voice playback (13.4 ± 6.6 s) than after an unfamiliar voice playback (82.5 ± 23.3 s, Wilcoxon signed-rank test: *T* = 7, *N* = 11, *P* = 0.037) (Fig. 1a). Moreover, when the stimulus was familiar cheetahs looked at the loudspeaker nearly five times longer than when it was unfamiliar (25.4 ± 8.4 vs 5.1 ± 2.0 s; Wilcoxon signed-rank test: *T* = 2, *N* = 11, *P* = 0.0058) (Fig. 1b).

Eight out of 11 individuals changed their activity after the familiar voice playback, whereas only two changed activity after the unfamiliar voice playback (Fig. 1c). The number of cheetahs who changed activity for the familiar voice and did not change activity for the unfamiliar voice was significantly higher than the reverse (Binomial test, *P* = 0.016). Changing of activity consisted in stopping walking for those that were walking at the time of the playback or standing up and starting walking for those who were sitting or lying down. The latency of activity change (involving a change in posture and/or locomotor movement) was 53 ± 19 s after the playback of the familiar voice and 131 ± 14 s after the playback of an unfamiliar stimulus. Cheetahs changed their activity three times faster when they heard a familiar voice than when they heard an unfamiliar voice (Wilcoxon signed-rank test: *T* = 0, *N* = 11, *P* = 0.018) (Fig. 1d).

## Discussion

Our results demonstrate that cheetahs are clearly able to discriminate between familiar and unfamiliar human voices with the former eliciting higher levels of visual attention and more frequent behavioural changes. These findings provide evidence that non-domesticated felids can use human voices as a basis for assessing familiarity with humans.

The first response of our tested individuals was visual attention, which is typical in studies involving playback experiments^28,35^. However, controversies are found in the available literature in the intensity of the response given to familiar *vs* unfamiliar stimuli. In some studies, animals showed a stronger visual attention (e.g. shorter latencies, longer gazes) towards familiar stimuli^7,11,36,37^, but the opposite was found in other studies^38–41^. Stronger visual attention to unfamiliar stimulus is typically found in violation of expectations paradigms. This is for example the case in the study of Sankey *et al*.^6^, the horse subject is not supposed to hear an unfamiliar human voice in the tested context, which explains that visual attention was stronger when hearing an unfamiliar human voice. At Cheetah Outreach, it is not unusual for cheetahs to hear voices of visitors, we are therefore not in a violation of expectation paradigm. The strong visual attention when hearing a familiar voice could be seen here as a mark of interest since the simulated handler may want to take out the animal or feed it. The cheetah had therefore probably learned to recognize the familiar voice through associative learning. In line with that, we found that our subjects were not only looking in the loudspeaker direction but they were also being more active (stand up or walk) when hearing a familiar voice, highlighting their motivation. This presents potential evidence of an anticipation of the incoming events such as food or change of enclosure.

These capacities and modalities of responses are similar to those observed in domestic felids, i.e. domestic cats^28^. Indeed, cats in this study reacted mainly with visual attention (and not vocally) as did the cheetahs in our study. While the authors argue that this ability in domestic cats is probably driven through the domestication process, our results suggest that this is not the only possibility. Cheetahs at Cheetah Outreach were born in captivity, bottle-fed and raised by humans and they have therefore developed a bond with humans. Moreover, choosing the two extremes categories of humans’ voices, and choosing the supposedly favorite handler for each cheetah allowed us to assume that the difference between the two types of relationships is as extreme, thus exacerbating the incremented response. The experience with humans, here mostly positive (feeding), without a process of domestication, has been therefore sufficient to allow this recognition capacity by a non-domesticated animal. It would now be interesting to investigate this question in another facility, where cheetahs’ experience with humans is more neutral.

This is the first evidence that non-domesticated felids are able to discriminate between familiar and unfamiliar humans, especially from acoustic cues. We can conclude from our study that domestication is not the only process that drives a fine scale recognition ability of humans. The proximity of these cheetahs with humans since an early age may have made human voice more salient for them. Furthermore, it is one of the first evidence that wild animals are able to distinguish between different types of human voices. Indeed, McComb and her colleagues^21^ have already shown that elephants could discriminate between different ethnicity, gender and age of people on the basis of their voice, but our study is the first to our knowledge involving an individual scale recognition on the basis of the voice in wild animals. Investigating such abilities through cross-modal recognition could be the next step for eliciting the impact of proximity and early experience of an individual in improving its discrimination capacities of humans. Another implication in light of the ability to discriminate between familiar and unfamiliar human voices is that we could expect that cheetahs are actually able to discriminate between familiar and unfamiliar conspecifics on the basis of acoustic cues. Indeed, several species encode individuality in their vocalizations and are able to recognize individuals by their calls^42–45^. Potential to encode individual identity in their vocalizations was also reported for cheetahs^30^ and our study suggests that the mechanisms and cognitive capacities that allow the auditory recognition of individuals are present in cheetahs as well and exploring more deeply the vocal communication of cheetahs could help us better understand it as well as deepen our knowledge about the evolution of vocal communication.

### Compliance with ethical standards

All applicable international, national, and/or institutional guidelines for the care and use of animals were followed. The study has been conducted in accordance with the current laws in France and in South Africa (agreement with the 2010/63/UE). The animal park staff was responsible for all animal husbandry and care. Our experiments were evaluated as respecting the ethical rules and as based on non-invasive observations by the “Comité Rennais d’Ethique en matière d’Expérimentation Animale” (i.e. Rennes Ethical comity for experiments using animals; CREEA approval #201806081359001).
